# Antisepsis

**Published:** 1890-01-11

**Authors:** 


### ANTISEPSIS.

Dr. Boeckel, of Strassburg, is an enthusiastic antiseptist, and has for years protested against prolonged drainage of wounds, but recently he has gone so far as to discard the process altogether, believing it to be positively erroneous. He has published a list of 36 operations, including the resections of the knee, with two amputations of the thigh, four of cancer of the breast, etc., in none of which was any drainage tube employed. In most the wounds were covered with iodoform dressings, which were left on undisturbed until the healing was complete. To minimise the risk of infection he directs his attention to the antisepsis of the instruments, and of every appliance that approaches the wound, washing his hands frequently during long operations in a weak sublimate solution, using pads of antiseptic gauze instead of sponges, and closing the cavity of the wound as far as possible by the employment of deep sutures. Dispensing with drainage tubes, he is generally able to effect perfect healing without a single repetition of the dressing, and in operations on the joints and bones he relies on elevation of the limb and compression of the arterial trunks for avoiding hæmorrhage in preference to ligatures and hæmorradic forceps, on which he looks as so many additional chances of septic infection. In none of these thirty-six cases was there any suppuration, fever, or appreciable pain, and repair was effected in a remarkably short time. In fact, he considers that drainage renders most antiseptic precautions more or less nugatory.

